# Frequency of missing teeth and reduction of mesiodistal tooth width in Japanese patients with tooth agenesis

**DOI:** 10.1186/s40510-018-0222-4

**Published:** 2018-08-20

**Authors:** Norihisa Higashihori, Jun-ichi Takada, Minami Katayanagi, Yuki Takahashi, Keiji Moriyama

**Affiliations:** Section of Maxillofacial Orthognathics, Department of Maxillofacial/Neck Reconstruction, Graduate School, Tokyo Medical and Dental University, Tokyo, Japan

**Keywords:** Hypodontia, Japanese, Oligodontia, Tooth agenesis, Mesiodistal tooth width

## Abstract

**Background:**

Tooth agenesis can involve one or more congenitally missing teeth (CMT) and is the most common congenital dental anomalies in humans. Tooth agenesis and reduction of mesiodistal tooth width are reportedly associated, suggesting that the pathogenesis of the two conditions is related. The current study analyzed the frequency of tooth agenesis and mesiodistal tooth width in cases of hypodontia (1–5 CMT) and oligodontia (≥ 6 CMT) in Japanese patients based on the hypothesis that reductions in mesiodistal tooth width are more frequently associated with oligodontia than hypodontia.

**Methods:**

Japanese patients with tooth agenesis were divided into hypodontia cases (60 female and 25 male, mean age 19.6 years, mean CMT number 1.31 ± 1.65) and oligodontia cases (26 female and 25 male, mean age 14.6 years, mean CMT number 8.07 ± 2.39). Controls included patients with a skeletal class I relationship and no CMT (female and 60 male, mean age 20.8 years). Dental casts and orthopantomograms were used to analyze the CMT frequency and mesiodistal tooth width for each group. The Kruskal-Wallis test, the Mann-Whitney *U* test, and Spearman’s rank correlation were used for statistical analysis.

**Results:**

In the hypodontia group, mandibular second premolars were the most frequently missing tooth type (25.9%), followed by mandibular and maxillary lateral incisors (19.4 and 17.1%, respectively). In the oligodontia group, mandibular second premolars were the most frequently missing tooth type (88.2%), followed by maxillary second premolars (87.3%) and first premolars (63.7%). In female subjects in the hypodontia group, only maxillary lateral incisors and mandibular first molars were significantly smaller than those of the female control subjects. In contrast, in the oligodontia group, more tooth types were significantly smaller than those of the control, for both sexes. Except for maxillary second premolars in female subjects, correlations were apparent for all tooth types in both sexes.

**Conclusions:**

Compared to hypodontia, more tooth types exhibited reduced mesiodistal tooth width in oligodontia. Correlations between CMT number and mesiodistal tooth width support the hypothesis that reduction of mesiodistal tooth width are more frequently observed in Japanese oligodontia patients than in Japanese hypodontia patients.

## Background

To obtain functional and esthetic occlusions, a harmonious relationship between the maxillary and mandibular teeth is essential. Disproportion of mesiodistal tooth width and tooth number induce an unharmonious relationship such as crowding or spacing in the dental arch, which clinicians must consider when making treatment plans, especially in orthodontic treatment. In such circumstances, an understanding of dental anomalies with respect to mesiodistal tooth width and tooth number is important for obtaining proper interdigitation, overbite, and overjet. Uslu et al. and Thongudomporn and Freer reported that the prevalence of having at least one dental anomaly in orthodontic patients were 40.3 and 74.8%, respectively [1, 2]; thus, patients with dental anomalies are commonly encountered in orthodontic clinics. Tooth agenesis is one of the most commonly observed dental anomalies. A meta-analysis found that its prevalence differed depending on race but averaged 6.4% [3]. In the Japanese populations, the prevalence of tooth agenesis is 8.5% [4]. While having one or two congenitally missing teeth (CMT) is common, having six or more is very rare, with prevalence less than 1% [4–8]. Reportedly, the most common CMT types are mandibular second premolars, maxillary lateral incisors, and second premolars in that order [3, 9].

The etiology of tooth agenesis is considered to involve the disturbance of dental development by genetic factors, environmental factors, or a combination thereof [10–16]. Tooth agenesis is often accompanied by other tooth anomalies [17–24]. Many researchers have reported that tooth size in patients with tooth agenesis is often smaller than in patients without tooth agenesis [19–24]. Gunor et al. reported that mesiodistal tooth width in patients with two or more CMT was significantly smaller than that of patients without CMT. Furthermore, reduction of mesiodistal tooth width was more severe in patients with six or more CMT than it was in patients with two to five [22]. Similarly, Brook et al. reported that there was a positive correlation between the degree of reduction of mesiodistal tooth width and the number of CMT [20]. However, these results were derived from Turkish and Caucasian populations—no reported studies have investigated the relationship between CMT number and mesiodistal tooth width in Japanese subjects. Because it has been reported that mesiodistal tooth width varies between races [25–27], it is considered clinically important to clarify the relationship between CMT number and mesiodistal tooth width in Japanese subjects.

In this study, we analyzed the relationship between the number of CMT and mesiodistal tooth width in Japanese hypodontia (1–5 CMT) and oligodontia (≥ 6 CMT) patients based on the hypothesis that reductions of mesiodistal tooth width are more frequently associated with oligodontia than hypodontia.

## Methods

### Patients

A total of 136 Japanese non-syndromic tooth agenesis patients in our hospital were divided into two groups, a hypodontia group consisting of patients with 1–5 CMT (*n* = 85; 60 female and 25 male, mean age 19.6 years, mean CMT number 1.31 ± 1.65) and an oligodontia group consisting of patients with ≥ 6 CMT (*n* = 51; 26 female and 25 male, mean age 14.6 years, mean CMT number 8.07 ± 2.39). A control group was also included, consisting of patients with a skeletal class I relationship and no CMT (*n* = 120; 60 female and 60 male, mean age 20.8 years). An a priori power analysis was conducted with G*Power Version 3.1 (Heinrich-Heine-Universität, Düsseldorf, Germany) to determine the sample size with effect size *f* = 0.25, *α* = 0.05, and 1 − *β* = 0.8 [28]. The tooth agenesis was diagnosed via panoramic radiography and clinical examination. The third molars were excluded from this study, as were the teeth with dental caries or restoration on the mesial or distal surface, non-congenitally missing teeth, and teeth that were not fully erupted. The frequency of missing tooth type was calculated as follows:

$$ {\displaystyle \begin{array}{l}\mathrm{Frequency}\ \mathrm{of}\ \mathrm{missing}\ \mathrm{tooth}\ \mathrm{type}\ \left(\%\right)=\left(\mathrm{total}\ \mathrm{number}\ \mathrm{of}\ \mathrm{missing}\ \mathrm{teeth}/\left(\mathrm{sample}\ \mathrm{number}\times \kern0.37em 2\right)\right)\ \\ {}\times 100\end{array}} $$

### Measurements of mesiodistal tooth width

Mesiodistal dimensions were measured on dental casts with a digital caliper (Mitutoyo, Kanagawa, Japan). Each measurement was performed twice on each tooth, and if the discrepancy was more than 0.4 mm, the measurement was repeated. The mesiodistal distance was defined as the greatest distance between the contact points of the tooth crown. One month later, 30 dental casts were randomly chosen, and the measurements were redone by the same examiner. There were no significant differences between the first and second measurements, as determined by the Wilcoxon signed-rank test.

### Statistical analysis

The Kruskal-Wallis test and the Mann-Whitney *U* test were used to compare differences between the groups. Spearman’s rank correlation was used to examine correlations derived from the measurements.

All procedures were performed with commercial statistical software (SPSS Release 13.0, Chicago, IL, USA). All tests were two-tailed, with *p* < 0.05 considered to be statistically significant.

### Ethics approval

All procedures in this study were approved by the Ethics Committee of Tokyo Medical and Dental University (No. 419) and complied with the Code of Ethics of the World Medical Association (Declaration of Helsinki).

## Results

### Frequencies of missing tooth types in the hypodontia and oligodontia groups

**Fig. 1:**
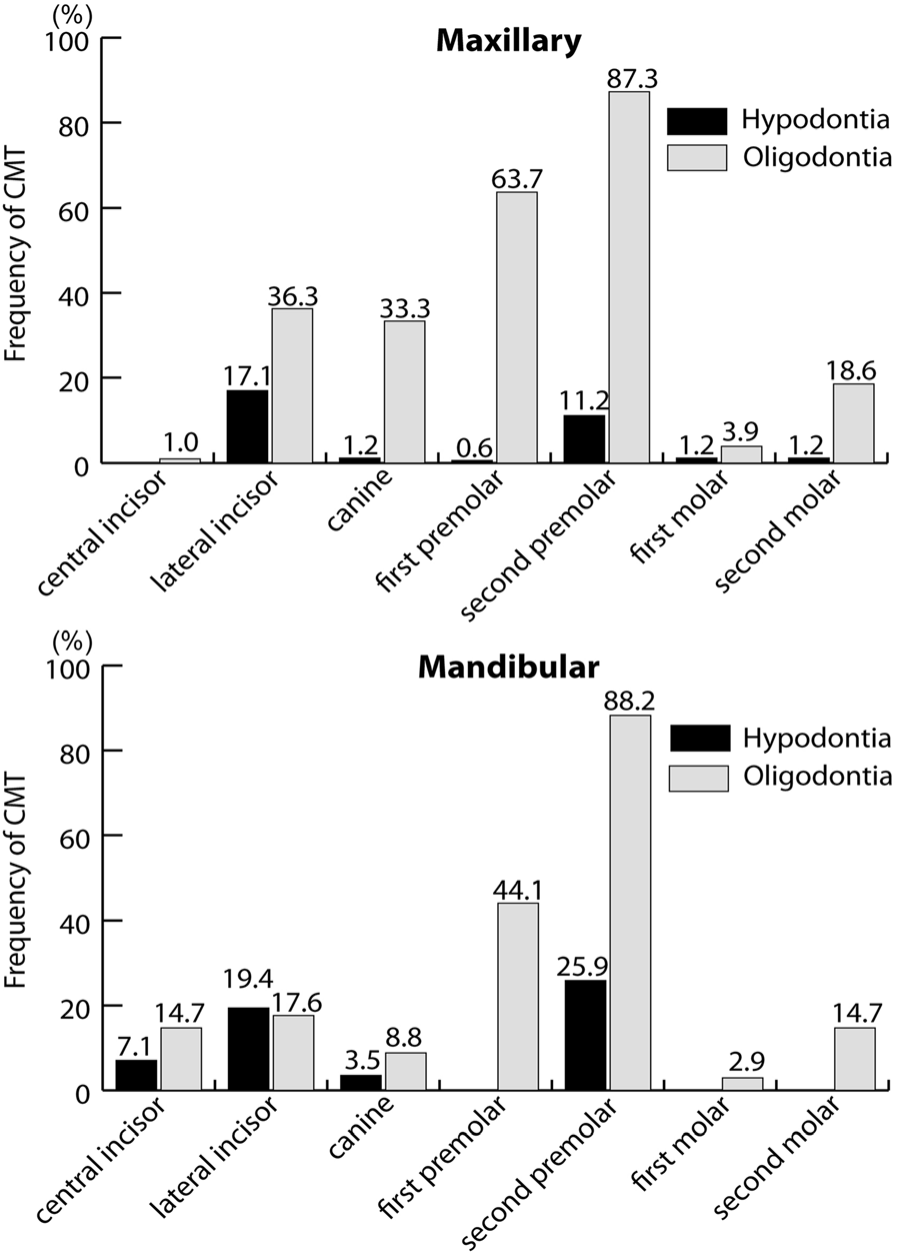
Frequency of congenitally missing tooth type in Japanese tooth agenesis patients

In the hypodontia group, lower second premolars were the most frequently missing tooth type (25.9%), followed by lower lateral incisors (19.4%) and upper lateral incisors (17.1%). In the oligodontia group, the most frequently missing tooth type was also lower second premolars (88.2%), but the frequency was much higher than it was in the hypodontia group. Unlike the hypodontia group, in the oligodontia group, the second and third most frequently missing tooth types were upper second premolars (87.3%) and upper first premolars (63.7%) (Fig. 1).

### Reduction of mesiodistal tooth width

In order to determine whether mesiodistal tooth width exhibited sex-dependent differences in our sample, we compared the mesiodistal tooth width of each tooth type in female and male patients. Because 7 out of 12 tooth types differed significantly depending on sex (data not shown), and it has previously been reported that tooth size is larger in male subjects, separate comparisons of tooth size were performed for male and female patients.

Only maxillary lateral incisors and mandibular first molars exhibited statistically significant mesiodistal reductions in the female hypodontia group in comparison to the female control group, and though they were statistically significant, the differences were small, − 0.35 and − 0.37 mm, respectively. There were no significant differences in mesiodistal tooth width in the male hypodontia group compared with the male control group. In contrast, in the oligodontia groups of both sexes, numerous tooth types were significantly smaller than those of the corresponding gender-matched controls. The differences in mesiodistal tooth width between the oligodontia patients and the controls were largest in the upper lateral incisors, − 1.97 mm in male patients and − 1.23 mm in female patients. In the oligodontia group, the tooth types that exhibited significant reductions of more than 0.50 mm compared to the control group were the maxillary central incisors (− 0.64 mm), maxillary lateral incisors (− 1.23 mm), mandibular lateral incisors (− 0.54 mm), and first premolars (− 0.56 mm) in female patients, and the maxillary central incisors (− 0.59 mm), maxillary lateral incisors (− 0.97 mm), first premolars (− 0.82 mm), first molars (− 0.61 mm), and mandibular first premolars (− 0.50 mm) in male patients (Table 1).

**Table 1:** Tooth size reduction in tooth agenesis Japanese patients

	Control (C)	Hypodintia (H)	Oligodontia (O)	C vs H		C vs O		H vs O	
	Mean ± S.D.	Mean ± S.D.	Mean ± S.D.	Difference	Significance	Difference	Significance	Difference	Significance
Male									
Maxilla									
Central incisor	8.81 ± 0.51	8.60 ± 0.62	8.22 ± 0.76	− 0.21	NS	− 0.59	**	− 0.38	*
Lateral incisor	7.40 ± 0.56	7.37 ± 1.00	6.43 ± 0.90	− 0.03	NS	− 0.97	**	− 0.94	**
Canine	8.38 ± 0.41	8.20 ± 0.56	7.94 ± 0.55	− 0.18	NS	− 0.44	**	− 0.26	*
First premolar	7.75 ± 0.44	7.77 ± 0.62	6.93 ± 0.40	0.02	NS	− 0.82	**	− 0.84	**
Second premolar	7.22 ± 0.50	7.13 ± 0.54	6.78 ± 0.46	− 0.09	NS	− 0.44	NS	− 0.35	NS
First molar	10.90 ± 0.69	10.99 ± 0.75	10.29 ± 0.75	0.09	NS	− 0.61	**	− 0.7	**
Mandible									
Central incisor	5.60 ± 0.37	5.79 ± 0.61	5.42 ± 0.39	0.19	NS	− 0.18	*	− 0.37	**
Lateral incisor	6.21 ± 0.43	6.21 ± 0.60	5.91 ± 0.50	0	NS	− 0.3	*	− 0.3	*
Canine	7.29 ± 0.39	7.16 ± 0.47	6.98 ± 0.46	− 0.13	NS	− 0.31	*	− 0.18	NS
First premolar	7.70 ± 0.37	7.69 ± 0.55	7.20 ± 0.47	−0.01	NS	− 0.5	**	− 0.49	**
Second premolar	7.64 ± 0.46	7.54 ± 0.46	7.13 ± 0.25	− 0.1	NS	− 0.51	NS	− 0.41	NS
First molar	11.76 ± 0.59	11.74 ± 0.71	11.32 ± 0.64	− 0.02	NS	− 0.44	**	−0.42	*
Female									
Maxilla									
Central incisor	8.69 ± 0.49	8.58 ± 0.49	8.05 ± 0.82	− 0.11	NS	− 0.64	**	− 0.53	**
Lateral incisor	7.29 ± 0.69	6.94 ± 0.89	6.06 ± 1.10	− 0.35	*	−1.23	**	− 0.88	**
Canine	7.98 ± 0.46	7.97 ± 0.49	7.76 ± 0.65	− 0.01	NS	− 0.22	NS	− 0.21	NS
First premolar	7.67 ± 0.43	7.51 ± 0.55	7.29 ± 0.58	− 0.16	NS	−0.38	*	−0.22	NS
Second premolar	7.08 ± 0.47	7.05 ± 0.57	7.20 ± 0.88	− 0.03	NS	0.12	NS	0.15	NS
First molar	10.62 ± 0.38	10.51 ± 0.60	10.35 ± 0.51	− 0.11	NS	− 0.27	*	− 0.16	NS
Mandible									
Central incisor	5.61 ± 0.37	5.66 ± 0.45	5.36 ± 0.57	0.05	NS	− 0.25	NS	− 0.3	*
Lateral incisor	6.19 ± 0.37	6.07 ± 0.50	5.65 ± 0.41	− 0.12	NS	− 0.54	**	− 0.42	**
Canine	6.86 ± 0.44	6.84 ± 0.49	6.60 ± 0.64	− 0.02	NS	− 0.26	NS	− 0.24	NS
First premolar	7.51 ± 0.43	7.46 ± 0.50	6.95 ± 0.56	− 0.05	NS	− 0.56	**	− 0.51	*
Second premolar	7.42 ± 0.44	7.34 ± 0.49	6.67 ± 1.17	− 0.08	NS	− 0.75	NS	− 0.67	NS
First molar	11.38 ± 0.48	11.01 ± 0.61	11.05 ± 0.89	− 0.37	**	− 0.33	NS	0.04	NS

*NS* not significant; *p < 0.05, **p < 0.01

### Correlations between CMT number and reduction of mesiodistal tooth width

We examined the correlation between CMT number and mesiodistal tooth width for each tooth type. Correlation analysis was performed with the datasets derived from the hypodontia and oligodontia groups pooled together. In female patients, there was a weak correlation for all tooth types except maxillary second premolars and mandibular first molars. In male patients however, correlations for all tooth types were stronger than they were in female patients, especially in the maxillary teeth (Table 2).

**Table 2:** Correlation between congenitally missing tooth number and tooth size reduction

	Male		Female	
	*ρ*	*p* value	*ρ*	*p* value
Maxilla				
Central incisor	0.43862	**	0.38505	**
Lateral incisor	0.47517	**	0.29453	**
Canine	0.52780	**	0.27117	**
First premolar	0.62379	**	0.22338	*
Second premolar	0.46537	**	0.00906	NS
First molar	0.55202	**	0.19545	*
Mandible				
Central incisor	0.38208	**	0.2475	**
Lateral incisor	0.48131	**	0.39227	**
Canine	0.38966	**	0.26579	**
First premolar	0.54793	**	0.36169	**
Second premolar	0.35016	**	0.36274	**
First molar	0.52197	**	0.09814	NS

*NS* not significant; *p < 0.05, **p < 0.01

## Discussion

Teeth develop from the dental lamina, then commence interactions with the epithelia and underlying mesenchyme. As tooth development advances, enamel knots mediate crown size and cusp formation [29]. Dental anomalies can occur due to disturbance of these processes by genetic factors, environmental factors, or both [10–16]. When unfavorable factors affect the initiation of tooth development, the tooth may not develop at all (tooth agenesis), whereas when they affect later development, they may result in changes in tooth morphology. Because the permanent teeth develop at different time-points, the influences of unfavorable factors can differ between tooth types. Later-developing tooth types in the same tooth family may have more of a chance of being exposed to unfavorable factors earlier in development. If the unfavorable factors exert influence early in the development of odontogenesis, such as at the time of initiation of tooth formation, there may be more of a possibility of severe effects such as tooth agenesis. If they exert influence at a later stage in development, the effects may be reduced and may only influence aspects of tooth morphology such as tooth size and cusp formation. Juuri and Balic [30] proposed that a gradual reduction in the odontogenic potential of dental lamina may explain why tooth agenesis most frequently affects the last tooth to develop within a tooth family. If the unfavorable effects on odontogenesis are weak, this gradual reduction in odontogenic potential may only affect the last tooth to form, as is apparent in some hypodontia patients. If the unfavorable effects are strong, however, more of the teeth may be affected by tooth size reduction and CMT. Concordant with these two ideas, later-forming teeth are much more susceptible to tooth agenesis and size reduction than earlier-forming teeth.

In this study, in the hypodontia group, mandibular second premolars were the most frequently CMT, followed by mandibular and maxillary lateral incisors, which is consistent with results reported by Endo et al. [4], who investigated 3358 Japanese orthodontic patients. Many researchers have reported that mandibular second premolars were the most frequently missing tooth type in tooth agenesis patients; however, in some reports, the maxillary and mandibular lateral incisors were the most frequently missing tooth type in Japanese [31, 32] and in other races [3, 9]. Regardless of the change in order, these teeth are the last teeth to form in their tooth family, which supports the contention that the last forming teeth are more susceptible to tooth agenesis. Although the mandibular second premolars were the most frequently missing tooth type in both the oligodontia group and the hypodontia group, there was a dramatic difference in frequency—25.9% (hypodontia) vs. 88.2% (oligodontia), and in the oligodontia group, the second, third, and fourth most frequently missing teeth were the maxillary second premolars (87.3%) and first premolars (63.7%) and mandibular first premolars (44.1%). These results are consistent with those of Endo et al. [4] and Ogaard et al. [33], which may indicate that an increased number of missing premolars is a typical feature of oligodontia patients.

Notably, in the current study, there was no significant difference in the reduction of mesiodistal tooth width in the hypodontia group except for maxillary lateral incisors and mandibular first molars in female, whereas other researchers reported a significant difference in mild tooth agenesis patients [17, 22, 34]. This may relate specifically to the fact that our sample population was Japanese, or it may relate to some other difference in sample collection. In the oligodontia group in the current study, however, as in the study of Gungor and Turkkahraman [22], in both sexes, reduction of mesiodistal tooth width was greater in cases of severe agenesis. Brook et al. [20] further concluded that as the number of CMT increase, the degree of tooth size reduction increases. The results of the current study are concordant with this in both sexes, with a higher correlation in male patients, particularly with regard to maxillary teeth. The details of CMT and reduction of mesiodistal tooth width pertaining to sex differences remain unclear at present, and further investigations are necessary in this respect.

Maxillary lateral incisors exhibited a higher frequency of CMT in both the hypodontia group and the oligodontia group in the current study. In a few reports, the frequency of tooth agenesis of maxillary lateral incisors was reduced in severe tooth agenesis patients compared to mild tooth agenesis patients [4, 5, 31]. In the current study, however, the frequency was still high. Moreover, maxillary lateral incisors exhibited the greatest difference in the oligodontia group, as has been reported by other researchers [20, 22]. Thus, it is possible that these teeth may be more susceptible to disturbances during development than other teeth because maxillary lateral incisors have simpler morphology, which may be more easily affected than that of any other teeth.

In contrast to the finding of high frequency of CMT in maxillary and mandibular second premolars in the oligodontia group, the mesiodistal tooth widths of these teeth, even in the oligodontia group, were not significantly differ from the sizes in the controls. These results differ from the findings of another study that maxillary and mandibular second premolars were also smaller in tooth agenesis patients [20, 22]. The discrepancy between the results of our study and their study may indicate that this phenomenon is specific to Japanese patients with tooth agenesis. However, our oligodontia patients included only four maxillary second premolars and three mandibular second premolars that were measured in both sexes, which suggests that larger numbers of teeth are needed to clarify this issue statistically. Further collection of samples is necessary to resolve this question.

The current study focused on the mesiodistal tooth width since it is believed that proper mesiodistal tooth width ratio of the maxillary and mandibular arches is necessary to have proper interdigitation, overbite, and overjet [35]. It is therefore important to know the characteristics of mesiodistal tooth width in tooth agenesis patients for making a treatment plan. However, because buccolingual dimensions and tooth shape were also affected in tooth agenesis patients [20, 22, 29], further analysis including three-dimensional analysis is essential to clarify the relation of tooth size/shape and tooth agenesis in Japanese patients.

### Limitation

All the samples, including the controls, were collected from Japanese orthodontic patients which limits the generalizability of our study. Richardson and Malhotra [36] and Johe et al. [35] reported tooth size discrepancies greater than ± 1 SD in 33.7 and 41% of their orthodontic patients, respectively. Our control samples also include disproportions in tooth size. However, the mean dimensions for the controls were similar to those reported by Brook et al. [20]. Nevertheless, because the samples were taken only from a Japanese population, the results should not be extrapolated to other racial population.

## Conclusions

In oligodontia patients, more varieties of tooth types showed reduced tooth size, and there was a correlation between CMT number and tooth size. These results support the hypothesis that tooth size reductions are more frequently observed in oligodontia cases than in hypodontia cases in Japanese patients.
